# The Effect of Water Content on Engine Oil Monitoring Based on Physical and Chemical Indicators

**DOI:** 10.3390/s24041289

**Published:** 2024-02-17

**Authors:** Fanhao Zhou, Kun Yang, Ling Wang

**Affiliations:** 1 Reliability Engineering Institute, School of Energy and Power Engineering, Wuhan University of Technology, Wuhan 430063, China; zfhwhut@163.com; 2 National Engineering Research Center for Water Transport Safety, Wuhan University of Technology, Wuhan 430063, China; 3 National Centre for Advanced Tribology at Southampton (nCATS), School of Engineering, University of Southampton, Southampton SO17 1BJ, UK; ling.wang@soton.ac.uk

**Keywords:** oil oxidation, water content, electrical conductivity, dielectric constant, acid number

## Abstract

Engine oil oxidation is one of the major reasons for oil aging which can result in variations in the physical and chemical properties of oil. Organic acids generated by oil oxidation can react with water to form inorganic acids and acidic substances (including organic and inorganic acids) that corrode engine parts, resulting in the generation of rust or damage to engine parts. This is one of the important reasons why oil should be regularly changed. One of the most commonly applied methods for judging the aging degree of engine oil is monitoring its acid number (AN). However, generally, the effect of oil water content on acid value measurement is not considered. When oils are used in engines, they are often contaminated by water due to condensation, which accelerates engine oil aging. Therefore, it is crucial to explore the water content effect on AN in the process of engine oil aging. In this research, a water content sensor was applied to characterize moisture content in oxidized oil samples. The sensor could also obtain oil sample electrical conductivity which corresponded to its dielectric constant. Using a mid-infrared spectrometer to measure oil sample AN at this point to obtain the variation in AN with oxidation time, oil sample AN was connected in series with the water content, dielectric constant and electrical conductivity. These parameters were monitored through sensors, and the effect of water content on AN was studied. Experimental results revealed that with the increase in oxidation time, the water content, electrical conductivity, dielectric constant increase and AN of oil were increased. At the same time, since the temperature had a greater effect on electrical conductivity, the application of an air-conditioned constant-temperature environment removed the effect of temperature change on electrical conductivity.

## 1. Introduction

**Figure 1:**
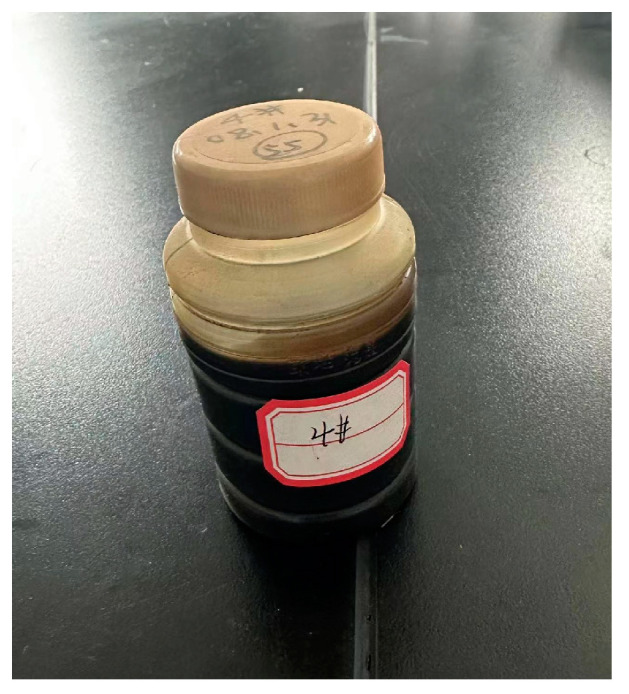
Aged engine oil.

Engine oils are exposed to high-temperature and high-pressure operation and are often in contact with air. Over time, oils are oxidized as part of the aging process, which results in variations in oil properties [1]. Figure 1 shows the aged engine oil.

If aged oil continues to be used, it will exert negative influences on the lubrication, cleaning and anti-corrosion efficiencies of engine oil [2]. Furthermore, when engine oil is working, it is subjected to catalytic effects of various metals and gradually ages, resulting in carbon deposits, sludge and dissolution of other substances that can cause damage to the engine [3]. This phenomenon accelerates engine component wearing, poor lubrication and other effects, affecting engine life. Therefore, the oil needs to be regularly changed. Engine oil’s physical and chemical properties are important indicators for measuring its quality [4]. The properties to be considered are viscosity, flash point, acid number (AN), ignition point, etc. Among them, AN plays a key role in measuring oxidation degree [5].

Base oil is oxidized during operation. At low temperatures (below 120 °C), oxidation goes through four stages of free radical chain reaction, irreversible free radical chain growth, chain branching and free radical chain termination [6]. In the early stage, second phases or chain alkyl radicals are formed to react with oxygen to produce oxygen free radicals, and then, other hydrocarbons extract hydrogen from hydrogen peroxide and other free radicals. During hydrogen peroxide generation and accumulation, the oil oxidation process eventually terminates. The product of this process is carboxylic acid, which results in an increase in oil acidity [7]. The typical oxidation reactions are as follows:

(1) $$CH_{3}-\left(CH_{2}\right)n-CH_{3}+O_{2}\to CH_{3}\left(CH_{2}\right)COCH_{3}+CH_{2}O$$

(2) $$CH_{3}{\left(CH_{2}\right)}_{n}COCH_{3}+O_{2}\to CH_{3}{\left(CH_{2}\right)}_{n}CO_{2}H+HCHO$$

(3) $$CH_{3}{\left(CH_{2}\right)}_{n}-CHO_{2}H-CH_{3}\to CH_{3}{\left(CH_{2}\right)}_{n}-CH_{2}O-CH_{3}+O$$

In engine oil aging process, not only acidic substances are produced, but also a trace amount of water is generated [8], as illustrated in the following reaction:

(4) $${2CO}_{2}H^{*}\to {COO}^{*}+{CO}^{*}+H_{2}O$$

The most commonly employed AN monitoring methods include titration and infrared spectroscopy; however, these methods cannot consider the effect of oil water content on research results. Water can exert great effects on oil monitoring results. This research studied the relationship of oil trace moisture and AN, thereby judging the impact of engine oil water content and providing reference opinions for engine oil replacement. In this research, first, an experimental platform was established based on the LubCosH20II water content sensor. The oil sample was prepared by adding iron acetylacetonate to engine oil. Karl Fischer titrant was applied for the determination of oil sample water contents after different oxidation times. The electrical conductivity and permittivity of oil samples were measured by the LubCosH20II sensor. Finally, an infrared spectrometer was applied for the verification of oil sample AN. Based on the correlation of the variations in water content and acid number with oxidation time, guidance is provided for research on oil change standards for engine oil.

## 2. Related Works

Several research works have focused on oil monitoring. Monitoring the variations in oil AN is an effective approach for engine oil condition monitoring. Kauffman et al. [9] measured total AN (TAN) using voltammetry techniques. Liao [10] applied the AD5933 Analyzer Module to develop a detection system for AN and achieve detection without chemicals. Ma [11] introduced a new method on the basis of mid-infrared spectral data and improved partial least squares (PLS) for the determination of the AN of used lubricating oil and developed multiple principal components of PLS. Chen [12] searched for a method for the detection of AN in transformer oil. The temperature titration method has also been used to determine the AN of transformer oil. Soleimani et al. [13,14] studied the feasibility of the application of thick-film (TF) sensors using ion-selective electrodes for the detection of lubricating oil aging. They found that thick-film electrodes presented a linear response to oil acidity variations at different temperatures, with electrical conductivity, viscosity and AN of oil samples increasing with the increase in oil oxidation degree. Yang et al. [15] further studied TF electrode reaction time in lubricating oil and the effect of lubricating oil moisture content on the detection performance of TF sensors. They found that glass-based TF sensors were able to work under high temperatures of up to 120 °C and metal particles in oil could help significantly decrease sensor response time. Wang [16] compared sensor output to TAN obtained with a widely accepted titration method. He found that the correlation between TAN and sensor output could be improved if the data scatter originating from the titration method was decreased. The original assessment was verified by measuring TAN using titration and electrochemical (EC) methods. Rivera-Barrera [17] concluded that the intensities and frequencies of oil mid-infrared attenuated total reflectance (MIR-ATR) spectra (4000–400 cm^−1^) could be applied as independent variables for several principal component regression (PCR) and partial least squares regression (PLSR) models. The latter was applied for the correlation of the spectra with their respective TAN values to construct a suitable prediction model. Twenty-six Colombian crude oil samples were used for the validation of the model. Beatriz [18] et al. measured TAN using infrared data and developed a prediction model to predict the degree of oil aging. Different techniques, including projection pursuit regression (PPR), partial least squares (PLS), support vector machines, linear models and random forest (RF), have been used. An innovative mechanism has been implemented for wider feature selection based on a genetic algorithm. Zhou [19] developed an acid value index prediction model based on an infrared spectroscopy monitoring method. The support vector machine regression method has been applied to quantitatively analyze oil sample AN, which verified the stability and prediction ability of the developed quantitative prediction model. In this work, we provide a theoretical basis and practical examples for online monitoring of oil indicators. Du [20] developed a new real-time quantitative detection method. After the first derivative pretreatment of spectra, discriminant analysis (DA) successfully identified cheap oil types adulterated in CAO with an accuracy of 96.7%. Macián [21] demonstrated that infrared spectroscopy is the most suitable method for quantifying diesel in used motor oil. Furthermore, the use of near-infrared spectroscopy in combination with a multivariate calibration approach allowed the prediction of fuel concentrations for samples used to validate the model.

It can be seen from relevant research works performed around the world that AN is an important indicator for engine oil monitoring. However, most researchers do not consider the effect of trace amounts of water on research results during the research process; therefore, errors may occur.

## 3. Experiment Preparation and Methods

### 3.1. Experimental Platform

**Figure 2:**
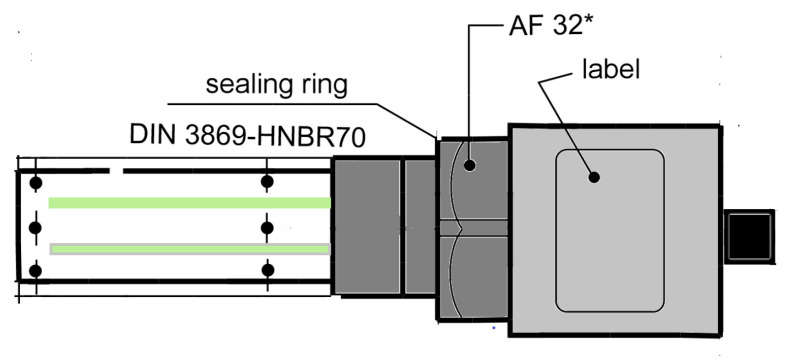
LubCosH20II sensor.

**Figure 3:**
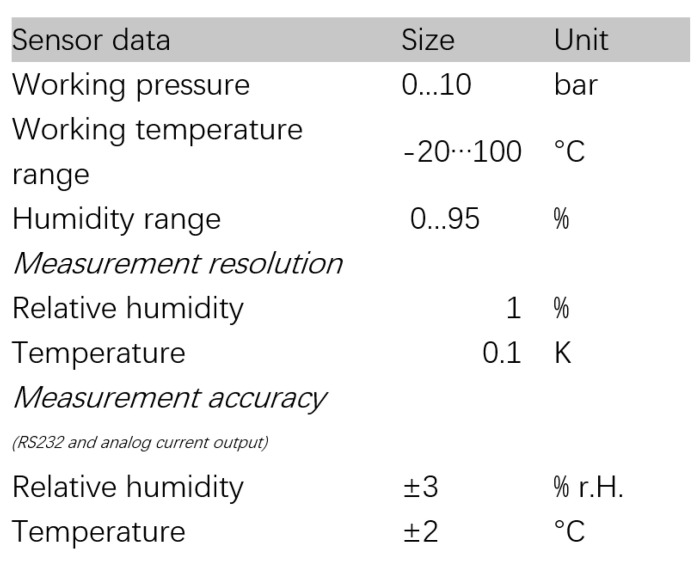
LubCosH20II sensor general data.

The LubCosH20II sensor (ARGO HYTOS, Karlsruhe, Germany) was applied for monitoring in this research. The sensing part was composed of a metal grid printed on a circuit board and two platinum metal wires without direct contact. Based on the measured electrical signal and the built-in circuit processing and conversion device, data transmission and exchange were performed directly with external equipment through a cable. The sensor detected the following physical characteristics of oil and their changes over time: temperature, relative oil humidity or relative dielectric constant (relative permittivity) and electrical conductivity of fluid. Since electrical conductivity and relative permittivity were strongly affected by temperature, the sensor also defined the values at a reference temperature (40 °C) in addition to characteristic values at the measurement temperature. The sensor automatically evaluated state changes. The structure and parameters of the LubCosH20II sensor are illustrated in Figure 2 and Figure 3.

### 3.2. Preparation of Oil Samples

In this research, base oil obtained from Great Wall Lubricants was artificially oxidized for the preparation of aged oil samples. Iron acetylacetonate is a catalyst capable of promoting oil deterioration. Iron acetylacetonate powder was added at a ratio of 0.3 g/50 mL to achieve a rapid testing process. Since oil’s flash point is 250 °C, in order to quickly oxidize the lubricating oil, it is generally heated at 70% of its flash point temperature; therefore, the heating temperature was set at 140 °C. Six groups of the same base oils were placed in round-bottom flasks of the same volume, and 0.4 g iron acetylacetonate was added as a catalyst to 50 mL of each of the six oil samples. An air pump was applied to blow air at a flow rate of 330 mL/min (air flow rate in cabin) into the oil sample while it was heated at a fixed temperature of 175 °C for 0, 2, 4, 8, 16 and 24 h.

Iron acetylacetonate powder is a β-diketone organic iron compound and is widely applied as a catalyst and ligand in organic synthesis. Acetylacetone can form stable complexes, especially with transition metal ions. These metal complexes can catalyze oxidation reactions and promote active redox reactions of oxygen molecules. At the same time, iron acetylacetonate provides an active surface capable of promoting oxygen adsorption and intermolecular reactions. In order to better and evenly oxidize the oil, air was introduced with a flow rate of 330 mL/min into the beaker of burning oil. The introduction of air resulted in some of the volatile components in the oil being evaporated and, at the same time, helped remove air bubbles from the liquid. Bubbles can affect liquid uniformity and stability, so blowing air in could remove bubbles and ensure a more uniform liquid. Furthermore, blowing air in could help mix and agitate the oil to ensure that its ingredients were evenly distributed.

The as-prepared oil samples were used for experiments. The oil sample information is shown in Table 1.

Experiments were designed for the verification of the catalytic effect of iron acetylacetonate powder. Using an electronic balance, 0.1–0.8 g of iron acetylacetonate (excessive powder would cause the oil to deteriorate) was weighed and added separately to sampled oils. Nine oil sample groups containing 0, 0.1, 0.2, 0.3, 0.4, 0.5, 0.6, 0.7, 0.8 g catalyst were prepared; air was passed through them at a flow rate of 330 mL/min, and they were heated at 175 °C for two hours. After cooling, the viscosity of samples was measured by a rotational viscometer, and the results are shown in Table 2.

It was seen that the addition of a catalyst had no effect on the viscosity index of oil samples. Therefore, the addition of iron acetylacetonate did not affect oil sample quality. Small errors could be generated due to too slow viscometer rotor rotation speed, resulting in an overly large range and making the obtained results less accurate.

Stabilization experiments were designed to select the optimum amount of iron acetylacetonate powder. The principle of this experiment was the measurement of electrical conductivity stabilization time. Methods with shorter monitoring times are needed. Shorter stabilization times for conductivity meant shorter times for data output. At the same time, on the basis of a stable range of electrical conductivity, it could be confirmed that the catalyst did not affect the physical and chemical indicators of oil samples.

**Figure 4:**
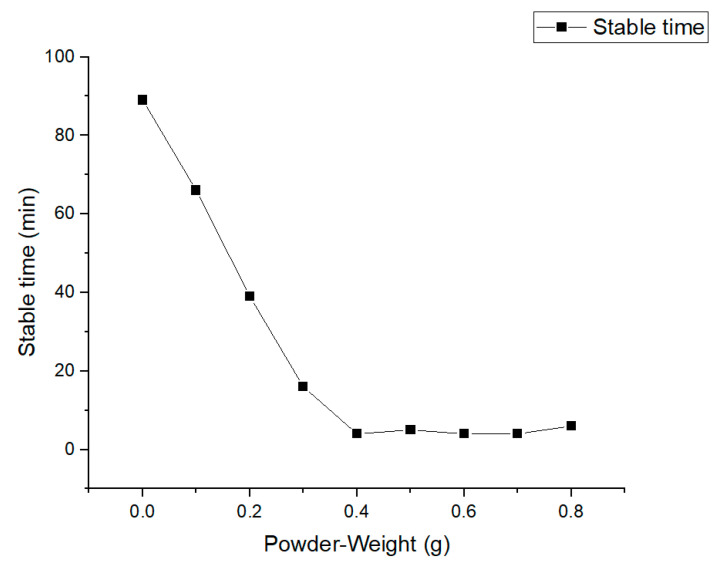
The relationship between catalyst amount and electrical conductivity stabilization time.

Sensors were applied to monitor the electrical conductivities of nine groups of oil samples, and electrical conductivity stabilization times were recorded. The obtained stabilization times are summarized in Figure 4.

It was seen from the obtained results that with the increase in the amount of catalyst iron acetylacetonate (accelerator), the stabilization speed of the curve showed a gradually accelerating trend. After the addition of 0.4 g catalyst, although there was still a slight speed change, the difference was small and could be ignored. Therefore, we selected 0.3 g as the optimal catalyst amount.

### 3.3. Sensor Calibration

**Figure 5:**
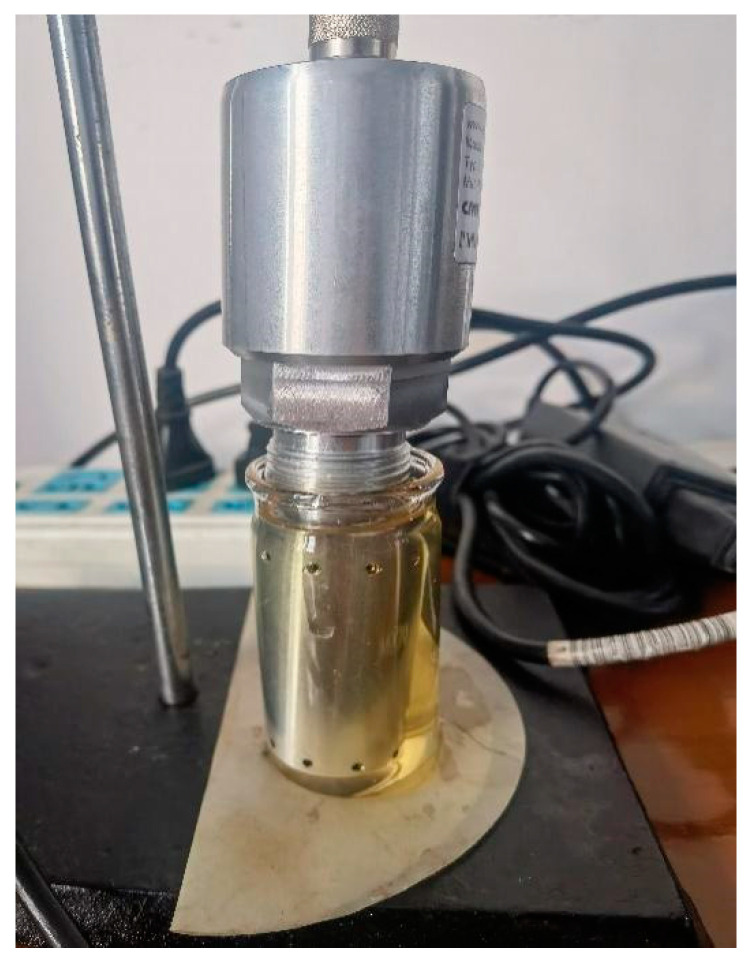
LubCosH20II sensing system.

In the beginning, the sensor system (as shown in Figure 5) had to be calibrated. A standard oil sample was applied as a calibration sample. The base oil was put into a 50 mL small beaker, and a sensor probe was placed into the beaker so that it was completely immersed in the oil sample.

**Figure 6:**
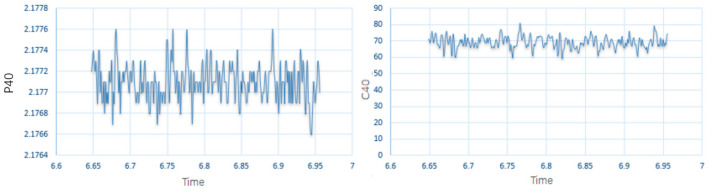
Dielectric constant (**left**) and electrical conductivity (**right**) of base oil.

Oil sample electrical conductivity and permittivity could be read by a computer connected to the sensor. The results measured using LubMonPClight software (Software Zoom PC-Visualization and Recording Software for Oil Sensors) are illustrated as Figure 6 (under the system-defined reference temperature, the dielectric constant was represented by P40, and the electrical conductivity was represented by C40).

**Figure 7:**
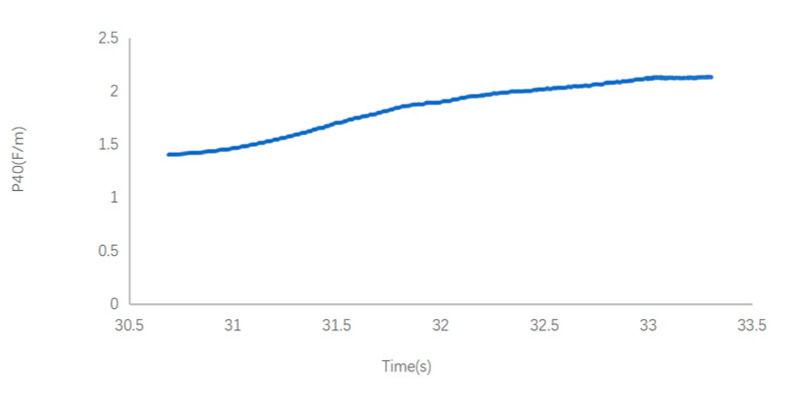

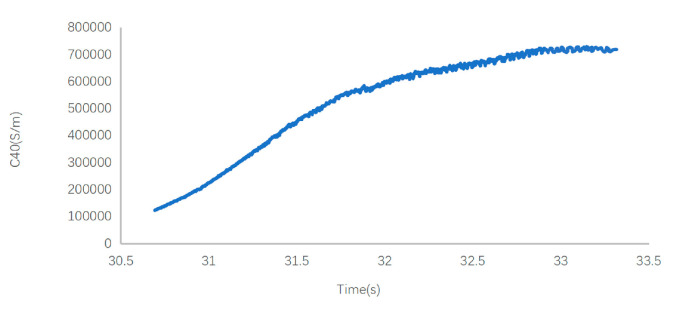
Dielectric constant (first) and electrical conductivity (second) of base oil (added water).

It can be seen from the figure that the electrical conductivity and dielectric constant of the base oil were dynamically stable within a certain range. Also, the stabilization time was short. Then, trace water was added to base oil samples for testing. The test results are shown in Figure 7

From the above curves, it can be seen that the oil sample electrical conductivity and dielectric constant were stable and the curve was smooth within a short period of time. This meant that the sensor was sensitive. Furthermore, there were no additional variables other than moisture difference.

### 3.4. Elimination of Temperature Effects

The oil environment temperature presented a certain instability and was slightly changed at different times. In order to explore whether the test results were affected by temperature at different experimental times, the following experiments were designed.

**Figure 8:**
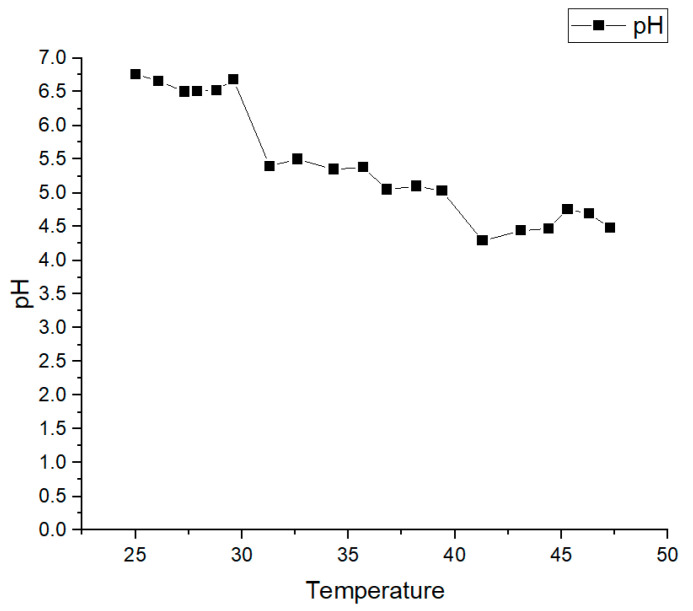
Changes in pH with temperature.

Using a temperature-controlled heating and stirring device, the same group of oil samples was heated and evaluated, and the oil sample pH was measured at different temperatures. The obtained results are summarized in Figure 8.

Since laboratory temperature was considered to be 25 °C, the default temperature of data monitored without heating was 25 °C. It was seen that when temperature was in the range of 25–30 °C, it had little effect on pH. With the increase in temperature from 25 to 27 °C, the pH was slightly decreased. This was because temperature affected the ion product in oil, while the concentration of positive true hydrogen ions remained unchanged. Then, the data re-increased slightly with the increase in temperature. This was because the oil only contained a very small amount of water. However, hydrogen ions in oil were generated mainly due to the ionization of organic acids and not water; therefore, it could be considered that temperature had no effect on the acidity at this stage. At temperatures above 30 °C, it was seen that the oil sample pH was significantly changed. Therefore, at temperatures greater than 30 °C, the experiments needed to be temperature-compensated. An air conditioner was applied as temperature compensation, and the temperature was fixed at 25 °C.

## 4. Results and Discussion

After five groups of oil samples (not considering base oil) were oxidized at a high temperature, their water contents were measured with Karl Fischer titrant. For accurate data, each group of oil samples was tested three times, and the average value of water content was reported in Table 3.

**Figure 9:**
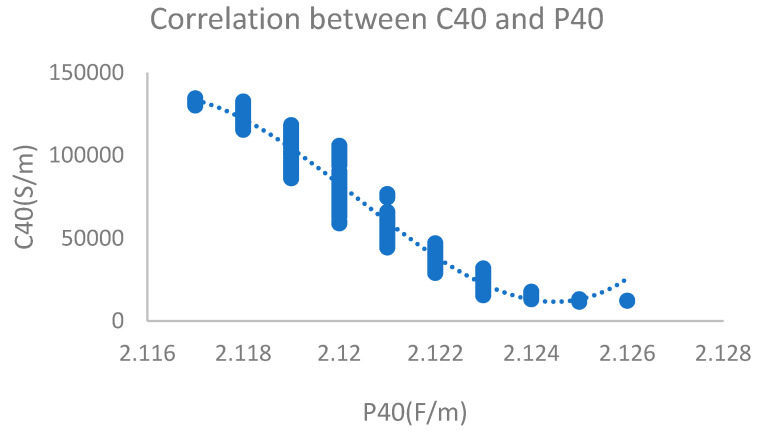
Dielectric constant and electrical conductivity with 120 ppm water content.

Using the built-up sensor system, when measuring an oil sample with 120 ppm water content (oxidation time of 2 h), the dielectric constant was 2.126 and electrical conductivity was 8974 PS/M, which finally reached a stable state. The dielectric constant and electrical conductivity showed a strong correlation, and curve fit (polynomial fit) results obtained from the least square method were also good, with a value of 0.93. The monitoring results are shown in Figure 9.

**Figure 10:**
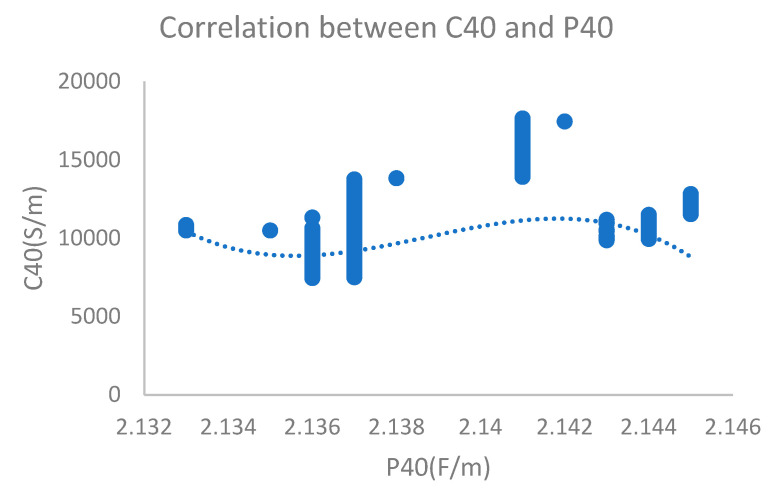
Dielectric constant and electrical conductivity with 139 ppm water content.

According to the test results obtained for an oil sample with 139 ppm water content (oxidation time of 4 h), the dielectric constant was 2.133 and electrical conductivity was 9994 PS/M, which was stable. There was no correlation between electrical conductivity and permittivity. The monitoring results are shown in Figure 10.

**Figure 11:**
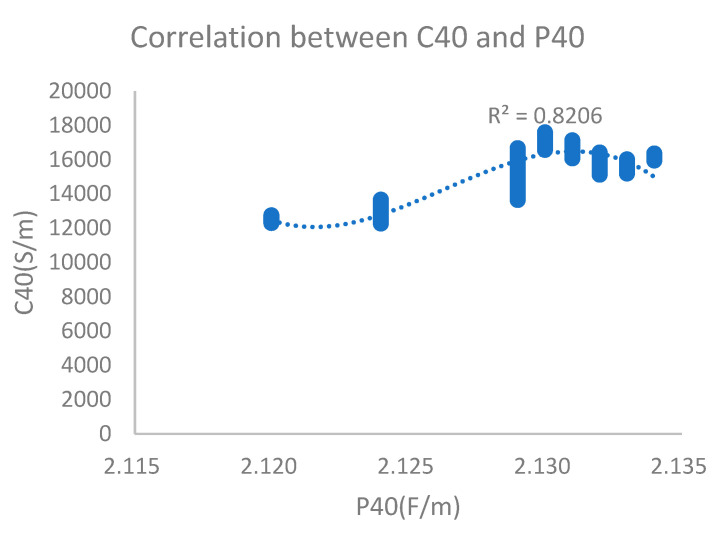
Dielectric constant and electrical conductivity with 161 ppm water content.

When an oil sample with 161 ppm water content (oxidation time of 8 h) was measured, the figure showed that the dielectric constant was 2.134 and electrical conductivity was 16,123 PS/M, which presented a correlation coefficient (polynomial fitting) of 0.82. The monitoring results are shown in Figure 11.

**Figure 12:**
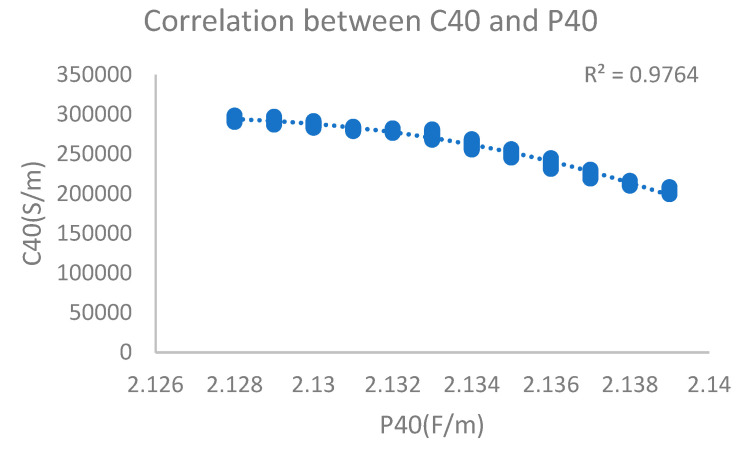
Dielectric constant and electrical conductivity with 182 ppm water content.

By measuring the oil sample containing 182 ppm water content (oxidation time of 16 h), it could be concluded that the dielectric constant was stable at 2.139 and the electrical conductivity was stable at 206,351 PS/M. For the relationship between electrical conductivity and dielectric constant, the curve-fitting polynomial obtained by the least square method was better, and the correlation coefficient reached 0.98, which had good reference significance. The monitoring results are shown in Figure 12.

**Figure 13:**
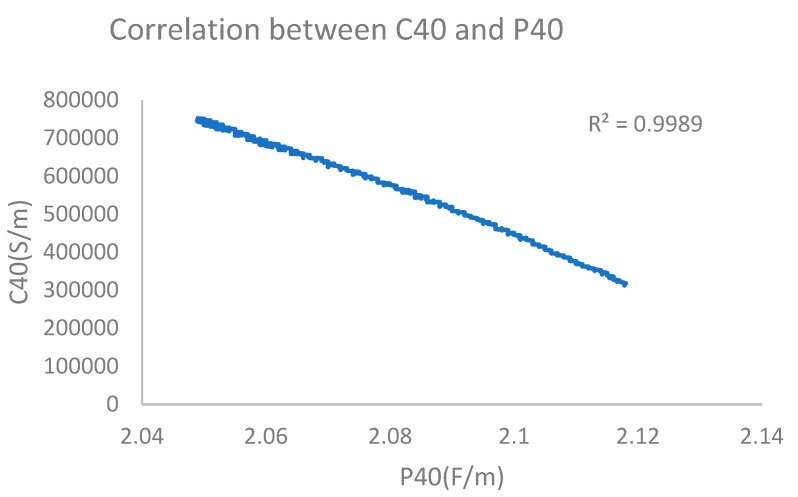
Dielectric constant and electrical conductivity with 201 ppm water content.

When an oil sample with 201 ppm water content (oxidation time of 24 h) was measured, the dielectric constant was stable at 2.118, and electrical conductivity was stable at 310,499 PS/M. At the same time, the curve fit between electrical conductivity and dielectric constant was relatively high. The correlation coefficient of polynomial fitting using the least square method reached 0.99. The monitoring results are shown in Figure 13.

For this group of experiments, the experimental results of the final five oil sample groups are shown in the Table 4.

From the table, it can be seen that at oxidation times of 2–16 h, the increase in water content first decreased and then increased the oil sample dielectric constant and increased electrical conductivity. This was because the increase in water content provided more free charged ions to the oil sample. At the same time, water existed in the oil in a dynamic and stable state. Both free water and mixed water were present. Therefore, the oil sample’s electrical conductivity was increased. As oxidation time was increased, engine oil produced more and more acid. Therefore, oil sample AN was increased. Since oil AN presented a good positive correlation with the dielectric constant, its dielectric constant was also increased. At an oxidation time of 24 h, the oil sample dielectric constant was decreased. This might be due to long-term high-temperature oxidation causing the evaporation and loss of water to a certain extent.

Furthermore, a horizontal comparison of the obtained experimental results revealed that the change laws of the dielectric constant and electrical conductivity were the same; that is, the dielectric constant and electrical conductivity had the same increasing and decreasing trends. This proved that there was a certain relationship between the dielectric constant and electrical conductivity. The horizontal comparison was mainly on a longitudinal span of time, which could explore the mathematical relationship between dielectric constant and electrical conductivity.

**Figure 14:**
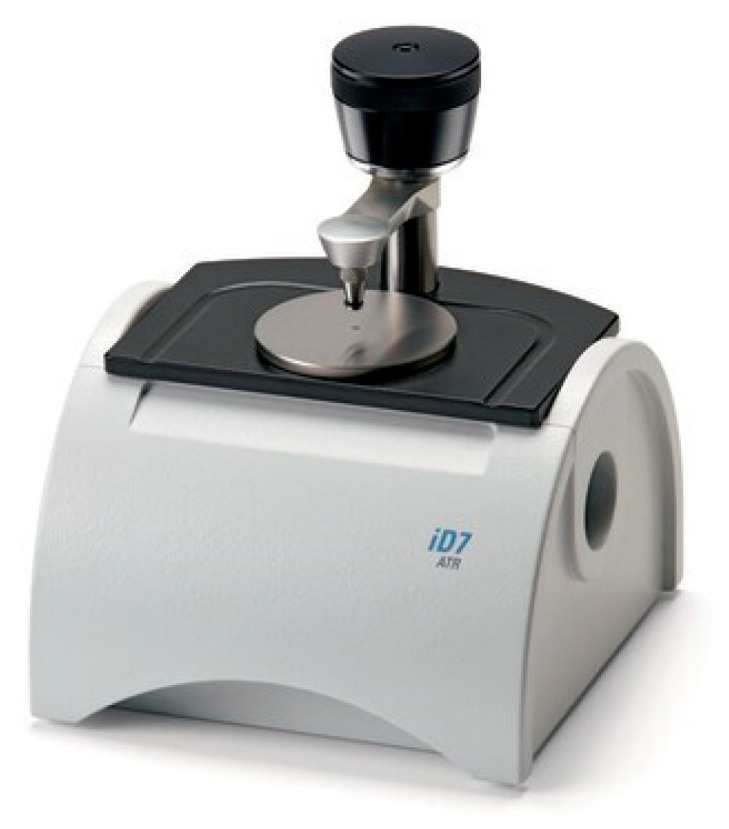
ATR accessories and schematic diagram.

It is known that there is a good positive correlation between the dielectric constant and AN in engine oil [22]. In order to better verify the relationship between water content and AN, mid-infrared spectroscopy was applied to determine the AN of the prepared oil samples. The reference standard method ASTM E2412 was adopted for measuring total AN by infrared spectroscopy. The infrared spectra of oil products were collected using a Fourier transform infrared spectrometer and attenuated total reflection accessories, using the collection and analysis software OMNIC developed by Nicolet. Figure 14 shows the ATR accessories used for infrared spectroscopy monitoring

Using OMNIC software, background spectra were collected before the collection of sample spectra in order to subtract the effects of moisture and carbon dioxide in the atmosphere. Then, a pipette was used to absorb a quantitative amount of the oil sample to be sided, which was dropped evenly on the ZnSe crystal of the attenuated total reflection accessory, and the collection of the spectrum of the oil was started.

**Figure 15:**
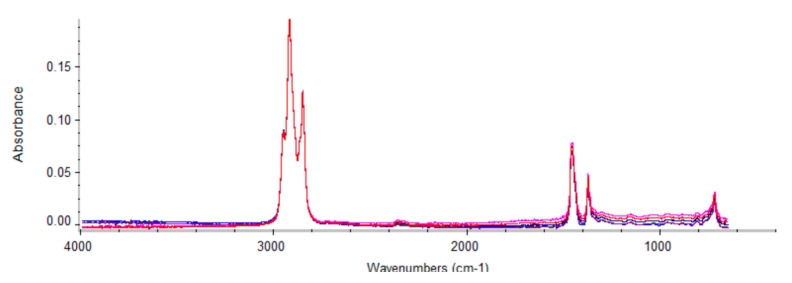
Spectral comparison.

The Figure 15 shows the spectral comparison of the six groups of oil samples.

Here, 3000~2500 cm^−1^ was chosen as the characteristic peak area for measuring oil sample AN. A hydroxyl O-H stretching vibration peak characterized the produced hydroxy acid. There was a coupling of O-H in-plane deformation vibration and C-O stretching vibration in the vicinity of 1420 cm^−1^ and 1300 to 1200 cm ^−1^. There were various interferences for O-H out-of-plane deformation vibration near 920 cm^−1^, or the absorption peak was not strong enough and was only used as a qualitative reference, not for accurate quantitative analysis.

**Figure 16:**
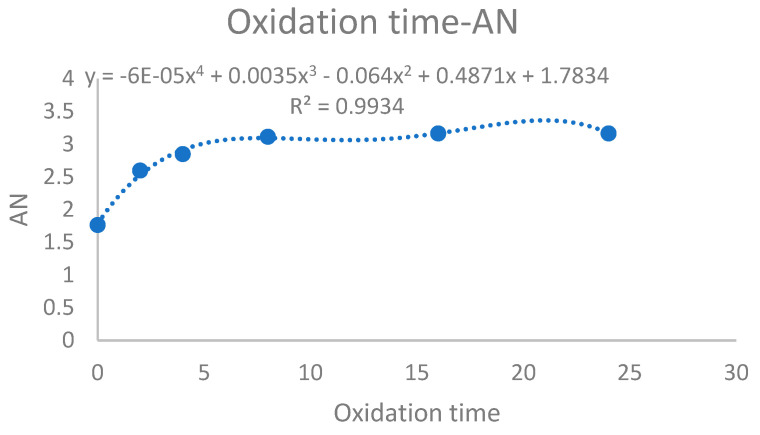
The relationship between oxidation time and AN.

Using the area method to characterize the ANs of the six groups of oil samples, the relationship between AN and oxidation time could be obtained, and is shown as Figure 16.

It can be seen from the figure that after 0–8 h, oil sample AN tended to increase. This was because acidic molecules were generated at this time, and acidic molecules decomposed under heating and reacted with alkane molecules in engine oil to produce acidic substances. After 8–24 h, the increasing trend of AN slowed down. This was because free H^+^ in oil samples was close to dynamic equilibrium.

This experiment verified that AN was increased with oxidation time. In other words, water content in oil samples was positively correlated with electrical conductivity, dielectric constant and AN. Therefore, changes in AN can be monitored through changes in moisture content. When AN reaches the critical value required by the oil, it means that the oil needs to be replaced.

## 5. Conclusions

In this research, the relationships between water content and electrical conductivity and between dielectric constant and AN were studied using a LubCosH20II sensing device. From the obtained scatter plot and fitting curve between electrical conductivity and dielectric constant, it was seen that the relationship between the variations in dielectric constant and electrical conductivity was a positive correlation. As lubricating oil oxidation time was increased, the correlation coefficient of the fitting equation was gradually increased, indicating that this method was not suitable for monitoring lubricating oil aging in the early stage of lubricating oil use. On the other hand, the fitting equation correlation coefficient between electrical conductivity and dielectric constant reached 0.82–0.99, indicating that mutual influence factors between electrical conductivity and dielectric constant were strong. AN could be characterized by the relationship between the water content, electrical conductivity and dielectric constant, which could be applied as a new method for monitoring lubricating oil quality. Experiments proved that an increase in oxidation time increased the oil water content, electrical conductivity, dielectric constant, and AN. In this research, the temperature effect was excluded using a constant-temperature method.

## Figures and Tables

**Table 1:** Experimental oil samples.

Time of Oxidation	ID	Quantity	Supplier
No. 40/0 h/air	R130003622	50 mL	Great Wall Lubricant
No. 40/2 h/air	R140000119	50 mL	Great Wall Lubricant
No. 40/4 h/air	R140000120	50 mL	Great Wall Lubricant
No. 40/8 h/air	R140000121	50 mL	Great Wall Lubricant
No. 40/16 h/air	R140000122	50 mL	Great Wall Lubricant
No. 40/24 h/air	R140000123	50 mL	Great Wall Lubricant

**Table 2:** Viscosity of oil samples containing different amounts of catalyst.

Number	Oxidation Time	Weight	Viscosity
1	2 h	0 g	212 mPa·s
2	2 h	0.1 g	223 mPa·s
3	2 h	0.2 g	209 mPa·s
4	2 h	0.3 g	197 mPa·s
5	2 h	0.4 g	213 mPa·s
6	2 h	0.5 g	208 mPa·s
7	2 h	0.6 g	203 mPa·s
8	2 h	0.7 g	202 mPa·s
9	2 h	0.8 g	203 mPa·s

**Table 3:** Water contents of experimental oil samples.

	Oxidation Time	2 h	4 h	8 h	16 h	24 h
Water content	Average value	120 ppm	139 ppm	161 ppm	182 ppm	201 ppm
	Average error	±1 ppm	±6 ppm	±3 ppm	±3 ppm	0

**Table 4:** Data of experiments.

Oil Sample	Water Content (ppm)	Dielectric constant (P)	Electrical Conductivity [pS/m]
1	120	2.126	8974
2	139	2.133	9994
3	161	2.134	16,123
4	182	2.139	206,351
5	201	2.118	310,499

## Data Availability

No new data were created or analyzed in this study. Data sharing is not applicable to this article.
